# Current Status of Physical Literacy in Japan: A Narrative Review of Research and Practical Applications

**DOI:** 10.14789/ejmj.JMJ25-0026-P

**Published:** 2026-02-17

**Authors:** KENTA TOYAMA, KOYA SUZUKI, MISAKI MATSUNAGA

**Affiliations:** 1 Graduate School of Health and Sports Science, Juntendo University, Chiba, Japan; 1 Graduate School of Health and Sports Science, Juntendo University, Chiba, Japan; 2 Institute of Health and Sports Science & Medicine, Juntendo University, Chiba, Japan; 2 Institute of Health and Sports Science & Medicine, Juntendo University, Chiba, Japan; 3 Juntendo Administration for Sports, Health, and Medical Sciences, Tokyo, Japan; 3 Juntendo Administration for Sports, Health, and Medical Sciences, Tokyo, Japan

**Keywords:** physical literacy, physical education, physical competence

## Abstract

In recent years, physical literacy has attracted increasing attention globally. Interventional studies have been conducted in several countries. However, there is still no established Japanese definition of “physical literacy.” In 2022, the Japan Sports Agency became the first government agency in Japan to discuss physical literacy from the perspective of improving children's physical fitness and establishing exercise habits. Consequently, conference presentations centered on physical literacy began to appear gradually. However, the extent of physical literacy research and utilization in Japan remains unclear. Therefore, this study investigates the progress of research on physical literacy in Japan to the present day. After assessing the current situation, we identified issues and considered how physical literacy could be utilized in Japan's physical education and sports settings in the future. Once the final version of the Japanese physical literacy assessment scale is completed, various studies, including intervention studies, can be conducted across a wide range of fields. It is hoped that physical literacy research will be implemented in society and will contribute to the maintenance and promotion of the health of the Japanese.

## Introduction

Physical literacy is defined as “the motivation, confidence, physical competence, knowledge and understanding to value and take responsibility for engagement in physical activities for life”^1)^. Ke et al. conducted a literature review on the definition of physical literacy and reported that the above International Physical Literacy Association (IPLA) definition is the most widely accepted worldwide^2)^. This concept was first proposed by Margaret Whitehead, a British researcher at the International Women's Sports Federation, in 1993. It has gained global recognition and attention in various fields including physical and sports education. The term “physical literacy” was already in use by the early 1900s, but Margaret Whitehead laid the groundwork for discussion with her own unique ideas in her doctoral dissertation, which led to its presentation at Congress^3, 4)^. Carl et al. conducted a systematic review of intervention studies and found that this research theme has undergone rapid development in recent years^5)^. Although its interpretation varies depending on a country's culture, it can safely be said that more countries may introduce the concept of physical literacy to evaluate physical education in the future. In fact, the MINEPS—the International Conference of Ministers and Senior Officials Responsible for Physical Education and Sport, which includes UNESCO members who are sports ministers and researchers—is held regularly, and physical literacy has been proposed as an important concept for achieving quality physical education^6)^. Meanwhile, the OECD's “Future of Education and Skills 2030” project defines “physical literacy” for 2030 with a focus on physical activity, though the desired outcome is broadly defined as a healthy and active life^7)^. Moreover, Lloyd et al. argued that physical fitness tests do not align with the goals of physical education and proposed the use of physical literacy as an alternative evaluation method for physical education^8)^. However, there have been few studies on physical literacy in Japan. The Japan Sports Agency (JSA) explained the importance of physical literacy for the first time in its Third Sport Basic Plan^9)^. While awareness of the term “physical literacy” is not as high as in other countries, the concept itself is present throughout Japanese educational culture, even without using that specific term. Therefore, the study investigates existing research and practical applications related to physical literacy in Japan to understand the current situation.

## Search strategy for Japanese papers on physical literacy

First, we searched for Japanese researchers and papers published in Japanese journals using the keyword “physical literacy.” Papers were searched using the CiNii electronic database. The phrase “physical literacy” was searched for in the title; in addition, since physical literacy has two Japanese translations, “*shintai* literacy” and “*shintaiteki* literacy,” these terms were also searched within the same database. Japanese publications up to July 29, 2025, were searched without any restrictions on countries. Additional studies were identified through manual searches and article reference lists. Papers with themes that differed from physical literacy or were duplicates were excluded. Finally, 32 papers were extracted; the results are listed in Table 1. We also compiled a table of online news articles, columns, and personal and company blogs (Table 2). The extraction method involved searching for “physical literacy” in English and Japanese using Google Search and Google News, respectively. Additional studies were identified through manual searches by experts. There was a sharp increase in the number of published papers in 2018. This may be related to the fact that major domestic sports stakeholders, namely the JSA, the Japan Sport Association (JSPO), and the Japan Sport Council (JSC), successively released official statements on physical literacy from 2018 to 2022. Regardless, considering the number of articles shown in Table 2, while there were a few articles with unique interpretations of the term, the awareness of physical literacy has been increasing.

**Table 1:** Selected papers related to physical literacy

No.	Title	First author	Year of release	Journal name	Category	Targets
1	An investigation of actual conditions concerning "shintai literacy" of Fukushima University students^11)^	Araya, S.	2005	Annual report of Fukushima University	Report	College students
2	Investigation of actual conditions concerning "shintai literacy" of Fukushima University students: comparison part 2 of the 1st and 2nd semester results in 2005^12)^	Araya, S.	2006	Annual report of Fukushima University	Report	College students
3	A new perspective on junior development: fostering physical literacy^13)^	Ito, S.	2010	Japan Journal of Studies in Athletics	Report	Junior athletes
4	A study of teaching for developments of "shintai literacy"^14)^	Kanke, R.	2010	Annual report of Fukushima University	Report	College students
5	The 2011 curriculum design of the SPERT Project^15)^	Tanigawa, S.	2011	Journal of Sport and Physical Education Center, University of Tsukuba	Journal article	College students
6	Seeking a model that integrates athlete development and lifelong sports: Canada's LTAD and Australia's FTEM^16)^	Ito, S.	2014	Bulletin of Studies in Athletics of JAAF	Report	Junior athletes
7	Canadian athletics' Long-Term Athlete Development (LTAD) program^17)^	Ito, S.	2016	Bulletin of Studies in Athletics of JAAF	Report	Junior athletes
8	Reconsidering the model for youth development in track and field: prospects for elementary school track and field in Japan in relation to physical literacy development^18)^	Ito, S.	2018	Bulletin of Studies in Athletics of JAAF	Report	Junior athletes
9	Kids' athletics and physical literacy: the philosophy of IAAF kids' athletics and its development in Japan^19)^	Kobayashi, H.	2018	Bulletin of Studies in Athletics of JAAF	Report	Children
10	Practice of kids’ athletics and physical literacy: how is physical literacy perceived in kids’ athletics?^20)^	Mori, K.	2018	Bulletin of Studies in Athletics of JAAF	Report	Children
11	Challenges for promoting a model for developing young athletes that combines competitive and lifelong sports: focusing on the current state of physical literacy in North America^21)^	Saotome, H.	2018	Bulletin of Studies in Athletics of JAAF	Original paper	Junior athletes
12	The role and importance of physical literacy in athlete development and physical education: a case study of Canada and its applicability in Japan^22)^	Saotome, H.	2018	Journal of Japan Society of Sports Industry	Original paper	Junior athletes
13	Youth sports development project in the United States: a case study of the Aspen Institute Project Play^23)^	Saotome, H.	2018	Journal of Japan Society of Sports Industry	Original paper	Junior athletes
14	Incorporating unstructured free play into organized sports^24)^	Barreiro, J.A.	2019	Strength & Conditioning Journal Japan	Translated paper	Junior athletes
15	Research on the approach to sports activities during the growth period: building an athlete development model-1st report^25)^	Japan Sport Association	2019	Japan Sport Association report on Sports Medicine and Science Research	Report	Junior athletes
16	Physical literacy for the older adult^26)^	Robert, E.P.	2019	Strength & Conditioning Journal Japan	Translated paper	Elderly people
17	Research on the approach to sports activities during the growth period: building an athlete development model-2nd report^3)^	Japan Sport Association	2020	Japan Sport Association report on Sports Medicine and Science Research	Report	Junior athletes
18	Research on the approach to sports activities during the growth period: building an athlete development model-3rd report^27)^	Japan Sport Association	2021	Japan Sport Association report on Sports Medicine and Science Research	Report	Junior athletes
19	Effect of wrestling on elementary school children’s physical competence and exercise habits^28)^	Kimura, M.	2021	The Japan Journal of Coaching Studies	Original paper	Junior athletes
20	Ethnography at the field site of a remedial education program for children with developmental disorders: exploring the need to develop physical literacy through enjoyable movement activities^29)^	Miyahara, M.	2021	Japanese Journal of Qualitative Research	Original paper	People with disabilities
21	Consideration of Margaret Whiteheadʼs concept of “physical literacy”: In the context of the trends of discussions in Japan^4)^	Mikami, J.	2021	Japanese Journal of Sport Education Studies	Original paper	Not applicable
22	Considering the significance of physical activity in an age of uncertainty: a proposal for the introduction of physical literacy education^30)^	Sugiura, Y.	2021	Journal of the Japan Academy for Health Behavioral Science	Original paper	Not applicable
23	Femoroacetabular impingement: why movement literacy matters^31)^	Terrell, S.L.	2021	Strength & Conditioning Journal Japan	Translated paper	Athletes
24	Definition and assessment of physical literacy in children and adolescents: a literature review^2)^	Ke, D.	2022	The Journal of Physical Fitness and Sports Medicine	Original paper	Children
25	The potential of navigation technology in ski learning: toward the development of physical literacy in skiing^32)^	Takahashi, K.	2022	First author	Lecture paper	Junior athletes
26	Physical literacy^33)^	Nakazato, K.	2023	Journal of the Research Institute for Sport Science, Nippon Sport Science University	Journal article	Not applicable
27	A study on the development of a Japanese version of the physical literacy scale: applicability to high school students in Japan^34)^	Inui, J.	2024	Japan Journal of Lifelong Sport	Original paper	High school students
28	A study on the process of physical literacy development throughout the school age: focus on adults with regular exercise habits^35)^	Inui, J.	2024	Japan Journal of Lifelong Sport	Original paper	Students
29	The role and potential of parents involved in their children's sports activities: case study of wrestling^36)^	Misu, A.	2024	Bulletin of Institute of Sport, Senshu University	Original paper	Athletes' parents
30	Examining the factor structure of the physical literacy for life self-assessment tool (PL4L) among Japanese adults and its relationship with the stages of change model for participation in regular physical activity^37)^	Matsunaga, M.	2025	Frontiers in Public Health	Original paper	Adults
31	Physical literacy in adolescents^38)^	Suzuki, K.	2025	Japanese Journal of Sport Education Studies	Original paper	Adolescents
32	A consideration of positioning physical literacy in school physical education: for the realization of well-being in school education^39)^	Takahashi, K.	2025	Kyushu Journal of Physical Education and Sport	Original paper	High school students

**Table 2:** Online news articles, columns, and personal and company's blogs on physical literacy

No.	Title	Author/Interviewee	Date of publishing	Source	Targets	URL/Link
1	The importance of physical literacy: an article by Takahiro Mori^40)^	Mori, T.	2/14/14	Gold Standard Laboratory	Junior athletes	http://goldstandardlabo.com/blog/2014/02/14/physical-literacy/
2	LTAD summary #5 chapter 3: physical literacy^41)^	Nakahara, T.	6/3/22	Personal blog post	Junior athletes	https://note.com/nakahara77/n/n5d20dd1270e6
3	The Japan Football Association releases “Japan's Way”: the "bible” is expected to stimulate discussion, both positive and negative^42)^	Takeuchi, T.	7/15/22	Gekisaka (Japanese Soccer News)	Junior athletes	https://web.gekisaka.jp/news/japan/detail/?363412-363412-fl
4	Overcoming children's reluctance to exercise requires motivation, knowledge, and habit formation^43)^	Toyama, K.	10/17/23	The Nikkei	Children	https://www.nikkei.com/article/DGXZQOUE035S40T01C23A 0000000/
5	Rethinking the future of physical education and sports: focusing on physical literacy^44)^	Matsuo, T.	12/14/23	Sasakawa Sport Foundation	General public	https://www.ssf.or.jp/knowledge/spi/35.html
6	Report on Professor Kobayashi's special class, “Kids' Athletics Experience Program”^45)^	Public Relations Dept.	7/16/24	Chuogakuin University Official Website	Children	https://www.cgu.ac.jp/news/nid00000629.html
7	Physical literacy: let's cultivate the strength to thrive in society^46)^	Koyama, K.	11/15/24	The Jomo Newspaper	Children	https://www.jomo-news.co.jp/articles/-/563801
8	Enjoy physical play—building habits from early childhood^47)^	Suzuki, K.	11/19/24	Medical Tribune	Children	https://medical-tribune.co.jp/kenko100/articles/?blogid=34&entryid=564970
9	Active cities in Europe: PACTE's initiatives^48)^	Homma, K.	12/13/24	Sasakawa Sport Foundation	Not applicable	https://www.ssf.or.jp/knowledge/sport_topics/202412.html
10	Junior high school students experience the Tokyo Marathon. Report on the “Mini Tokyo Marathon" in Shibuya Ward^49)^	Tokyo Marathon Foundation	12/23/24	RUNNING STREET 365	Junior high school students	https://runningstreet365.com/racereport/42753
11	Initiatives for kids in active cities^50)^	Homma, K.	1/16/25	Sasakawa Sport Foundation	Children	https://www.ssf.or.jp/knowledge/sport_topics/20250116.html
12	The family is the key to children's exercise habits^51)^	Abe, K.	4/3/25	The Nikkan Sports	Children	https://www.nikkansports.com/premium/sports/news/202504020000644.html
13	Habits at home that improve children's physical strength^52)^	Abe, K.	4/10/25	The Nikkan Sports	Children	https://www.nikkansports.com/premium/sports/news/202504080000561.html
14	Reforming extracurricular activities with children: “Renovation Quest Lab” envisions the “club activities of the future”^53)^	Fujita, S.	6/11/25	Sports for Social	Junior athletes	https://sports-for-social.com/sports/mirai-no-bukatsu/
15	Exercise and sports nurture children's minds^54)^	Okade, Y.	1/23/24	JAMMY, Inc.	Children	https://rodystore.jp/blogs/senmon/240123?srsltid=AfmBOopV2zz2Mw6-_3t3fIeGfk_DEHHuZ8UbzBYjj9fPsXAnyBrA2jkF
16	Early childhood experiences with “physical play” nurture a healthy body that will last into the future!^55)^	Suzuki, K.	11/29/22	Good Health Journal	Children	https://goodhealth.juntendo.ac.jp/sports/000305.html
17	Parents' physical literacy influences children's opportunities for exercise^56)^	Someya, Y.	3/22/23	Good Health Journal	Parents	https://goodhealth.juntendo.ac.jp/social/000317.html
18	What is physical literacy?^57)^	Suzuki, K.	6/29/24	NHK Educational	Children	https://www.sukusuku.com/contents/qa/310037

## Research and reports by domestic sports stakeholders

Among the three aforementioned organizations, the JSC was the first to release official documents. A survey was conducted on “Home Environments for the Acquisition of Physical Literacy in Children” (in Japanese) between 2018 and 2022. Within four years, an online survey regarding families' physical literacy was conducted nationwide with 1,000 parents of children aged 5-15 years. In 2020, in-depth interviews were conducted with eight women with children aged 5-15 years, who did not participate in sports. Based on the survey results, the “Japan Sport Council Information Strategy Project Report” (in Japanese) was compiled and made available online^10)^.

Second, as mentioned above, the JSA officially formulated its Third Sport Basic Plan on March 25, 2022. This statement explains physical literacy as follows.

*Through physical education and health classes, we aim to increase the number of children who enjoy physical activity and are familiar with exercise in their daily lives, and to foster the qualities and abilities (commonly referred to as “physical literacy”) necessary to continue engaging in physical activity and sports throughout their lives, thereby leading healthy and happy lives both physically and mentally*^9)^.

The above wording appears under the heading “Establishing Daily Exercise Habits and Improving Physical Fitness for Children and Youth” in the Third Sport Basic Plan. Establishing exercise habits can be considered synonymous with physical literacy, which was the subject of this study. Given this context, the Japanese government's announcement of the importance of physical literacy in official documents may have led to a full-scale launch of physical literacy research by researchers in various fields within Japan. Moreover, the JSA's project for “Promoting Physical Activity Habits from Early Childhood” (in Japanese), commissioned by Juntendo University, was implemented in 2023. The JSA invited applications from seven prefectures interested in promoting this project, and the research began with physical literacy as the keyword. Several articles highlighting its importance have been published in the Juntendo University's Good Health Journal website. Additionally, a survey conducted in 2023, the final year of this project, reported that parents' physical literacy influenced their children's physical activity habits^58)^.

Further, the JSPO established the Sport Medicine and Science Committee in 1960 to support the promotion of sports in Japan. Since its inception, the committee has conducted various research projects related to sports, medicine, and science. They started a multi-year research study in 2021 to develop “Evaluation Scales for Japanese Version of Physical Literacy,” which targeted a wide range of age groups, from children to older people. This research is ongoing and a report detailing the four years of research can be downloaded from the JSPO website^59-62)^. The report was published in four volumes over four years, and Table 3 presents its content. The report detailed regular meetings, research surveys, and verifications conducted by the team members. Consequently, a draft evaluation scale was completed by the end of 2023. Team members held regular meetings to implement this evaluation scale. These reports represent a comprehensive compilation of all the findings on physical literacy in Japan; hence, they have been consolidated into a single table. A Japanese version of the evaluation scale was created with reference to examples from other countries. The globally recognized definition of physical literacy can be broadly categorized into the two main frameworks proposed by Whitehead and Dudley^2)^. The IPLA, chaired by Whitehead, defines physical literacy as “the motivation, confidence, physical competence, knowledge, and understanding to value and take responsibility for engagement in physical activities for life^1)^.” The definition proposed by Dudley was updated as follows: “physical literacy is about building the skills, knowledge, and behaviors to help us lead active lives. It is holistic learning that occurs through movement and physical activity, and integrates physical, psychological, social, and cognitive capabilities^63)^”. Japan's evaluation criteria were developed with reference to Dudley's definition, which emphasizes the social domain. The reason for this can be found in the spirit of *Bushido*, the way of the warrior, which has been passed down since ancient times in Japan^64)^. At the core of *Bushido* are values such as “*gi*” (righteousness), “*yu*” (courage), and “*jin*” (benevolence). These were the standards by which the *samurai* lived their daily lives and made their decisions. *Samurai* were also expected to act for justice at all times, and face dangers and hardships with courage. At the same time, they are valued for their compassionate behavior toward the weak and their respectful treatment of their enemies. This not only elevated their personal character but also served as a moral foundation for Japanese society as a whole. Although Nitobe's principles of *Bushido* are not explicitly stated in the moral education content of elementary and junior high schools, many aspects align with *Bushido* values such as “respect for rules” and “honesty and sincerity.” For example, guidelines for implementing the course of study for elementary and junior high school physical education state, “In teaching physical education, it is necessary to appropriately guide students in moral education according to the content.” The guidelines organize what should be taught in each grade level in a table, and descriptions regarding “respect for rules” and “sincerity” can also be found^65, 66)^. Furthermore, these values are connected to the elements of physical literacy^60)^. Chiyosaburō Takeda, later Vice President of the JSPO, was influenced by his mentor F. W. Strange, a University of Tokyo English teacher. Takeda devised a new Japanese version, presenting seven items as “Kyogido”^67)^. They were created in association with *Bushido*^68)^. Qualities and precepts such as “Sports are a means to achieve the purpose of training mind and body. Therefore, engaging in sports for the sake of sports alone must be strictly avoided”; “Make fair play your principle”; “Obey the referee”; and “Always conduct yourself with courtesy” cover the spirit of *Bushido* and share common phrasing with elements of the JSPO physical literacy assessment scale^69)^. Thus, it is evident that the ethical norms Nitobe expressed in “*Bushido*” continue to permeate school education and sports even after the *samurai* class ceased to exist^70, 71)^. Additionally, it is also important to note that some researchers criticize Nitobe Inazō's “*Bushido*” for lacking historical awareness. It is noted that Nitobe was not an expert in Japanese intellectual or general history, and that what he discusses in “*Bushido*” is primarily his personal opinion and creative interpretation^72, 73)^. Abe points out that parts of the ideology in “Bushido” led the nation toward militarism, a harsh criticism, given that war and moral education are fundamentally incompatible^68)^. Despite such critiques, it remains true that Nitobe, born and raised in the *samurai* lineage, communicated to the world the idea that *Bushido* formed the foundation of Japanese morality, and that the moral education content in the Japanese curriculum guidelines shows many similarities to the seven virtues of *Bushido*. The Japanese Society for Moral Education comprehensively examines and analyzes moral education in Japan. In its proposal for future moral education, Bushido is highlighted as a traditional Japanese philosophy^74)^.

**Table 3-1:** Development of a physical literacy assessment scale^59-61)^

Volume	Publication year	Title	Subtitle	First author
1st report	2022	Introduction		Naito, H.
		Discussions on physical literacy	Discussions on physical literacy (PL) in other countries	Okade, Y.
			Physical literacy as seen in Japan's health and physical education curriculum guidelines	Okade, Y.
		Definition and assessment of physical literacy		Suzuki, K.
		Physical literacy and sports policy	Development of LTAD in Canada and physical literacy	Ito, S.
			Physical literacy initiatives in Australia	Morioka, Y.
			Survey on physical literacy in Japan	Kasuga, K.
		Conclusion		Naito, H.
2nd report	2023	Overview		Naito, H.
		Basic considerations on the development of an evaluation scale for physical literacy in Japan	Evaluation scales for physical literacy for children in other countries	Suzuki, K.
			School education and physical literacy in Japan	Okade, Y.
			Community sports and physical literacy in Japan	Matsuo, T.
		Survey on the current status of physical literacy in Japan	Factor structure and related factors of physical literacy	Suzuki, K.
			Survey on the current status of physical literacy among young people (high school students)	Aono, H.
		Proposal for a Japanese evaluation method for physical literacy for children		Suzuki, K.
3rd report	2024	Introduction		Naito, H.
		Survey on the actual state of physical Literacy in Japan	Factor structure and related factors of physical literacy among high school students	Suzuki, K.
			Comparative analysis of physical literacy by age group	
			Survey on the actual state of physical literacy among elementary and junior high school students (preliminary survey 1)	
			Survey on the actual state of physical literacy among elementary and junior high school students (preliminary survey 2)	
		Proposed physical literacy assessment scale and its correspondence to the course of study guidelines of elementary and junior high school		Toyama, K.
		Conclusion		Naito, H.

**Table 3-2:** Research on the development, verification, and dissemination of a Japanese version of the physical literacy assessment scale^62)^

Volume	Publication year	Title	Subtitle	First author
1st report	2025	Overview		Naito, H.
		Development of a physical literacy assessment scale for children (elementary and junior high school students)	Exploration of characteristics and factor structure of physical literacy in elementary and junior high school students according to grade level	Matsunaga, M.
			Preliminary survey of a physical literacy assessment scale for elementary school students in lower and middle grades	Suzuki, K.
		Sports instructor training curriculum and physical literacy	Relationship between physical education, health education, community sports, competitive sports, and physical literacy	Toyama, K.

Moreover, Jigoro Kano, the founder of judo, also known as the father of Japanese physical education, studied jujutsu, refined it in his own way, and established the spiritual path of martial arts, giving birth to judo in 1882. The philosophies of judo are “*Seiryoku-Zenyo*” (maximum efficient use of energy) and “*Jita-Kyoei*” (mutual welfare and benefit), which demonstrate how one's mind and body should be in relation to the society and those around them. *Seiryoku-Zenyo* involves using one's physical and mental abilities to the fullest extent possible to contribute positively to society. In contrast, *Jita-Kyoei* means cultivating a spirit of respect, gratitude, trust, and mutual support toward others and striving to create a prosperous world for not only oneself but also others^75)^. The philosophy of judo has had a profound influence on both Japanese sports and society as a whole, teaching Japanese the importance of spiritual values and the significance of growing together. Therefore, it is not surprising that Japan places importance on physical literacy in its social domain.

Another example of a measure announced by the government is as follows: The Tokyo Metropolitan Board of Education, the largest educational organization in Japan, formulated the Tokyo Active Plan for Students for 2022. This plan aims to help children improve their physical fitness while enjoying exercise and sports. In the “2022 Tokyo Metropolitan Survey on Physical Fitness, Motor Skills, and Lifestyle and Exercise Habits of Children and Students” report (in Japanese), Suzuki of Juntendo University contributed to the discussion section, noting that domestic boards of education mentioned the importance of physical literacy for the first time in official documents^76)^. This likely created an opportunity to spread the concept of physical literacy in Japanese education, similar to other countries.

## Studies and reports by university faculty members

The search identified several studies and reports published by universities. Araya et al. and Kanke, faculty members of the Department of Health and Exercise Science at Fukushima University, wrote reports on physical literacy^11, 12, 14)^. This is one of the earliest studies on physical literacy in Japan. However, they did not refer to overseas research; rather, they explained physical literacy as a concept developed independently through the curriculum at their university. Tanigawa et al. on the other hand, identified physical literacy as a key component of the physical education curriculum at Tsukuba University^15)^. Their report described physical literacy as a tool for cultivating theoretical knowledge and practical skills related to health, physical fitness, and physical activity through individual sports. Note that the term “physical literacy” used in the physical education curriculum at the University of Tsukuba is one of the themes covered in the Sports Literacy course, which is taken during the freshman year. The course aims to teach various fundamental movement skills, and its content is aligned with the physical literacy framework outlined in Canada's Long Term Athlete Development model^77)^.

Furthermore, Nakazato, a faculty member at Nippon Sports Science University, emphasized that physical literacy involves understanding and practicing the importance of maintaining high-level physical activity for health maintenance and promotion^33)^. Thus, the interpretations of physical literacy in these studies are centered mainly on the physical domain.

## Other noteworthy research

Two notable studies were identified using a search engine (Table 1). Sugiura and Higuchi addressed the improvement in physical fitness in humans, which has become more critical since the COVID-19 pandemic and suggested considering physical literacy rather than merely pursuing improvements in physical activity implementation or measurement values^30)^. Their study emphasized the importance of challenging the paradigm shift from traditional education led by exercise instructors and researchers to physical literacy education, which creates well-being in the community, with each individual playing a leading role. This research undoubtedly prompted the Japanese to consider how to utilize physical literacy, a familiar term in the nation.

Moreover, Miyahara et al. conducted a qualitative investigation into physical literacy in physical education for children with developmental disabilities, examining whether it is possible to foster not only motor skills, but also “enjoyment” and a “sense of accomplishment^29)^.” Given the limited number of studies on physical literacy in Japan, this is extremely valuable research that focuses specifically on children with developmental disabilities. As mentioned previously, the Japanese version of the physical literacy assessment scale for Children is under development, and this research may contribute to the development of a scale that can be utilized in special education classrooms in the future.

## Translated papers

All translated papers have been published in the *Strength and Conditioning Journal Japan*, the official journal of the National Strength and Conditioning Association (NSCA) Japan. The NSCA is a well-known nonprofit organization that focuses on advancing strength training and conditioning practices in the United States. Established as a Japanese branch, NSCA Japan had 10,640 members as of March 31, 2024, all of whom had access to the journal^78)^. NSCA certification requires the completion of a specific number of continuing education units (CEUs) every three years to maintain certification. This ensures that certified professionals remain current with the latest research, techniques, and best practices in terms of strengths and conditioning. Reviewing articles and passing a 10-question-quiz online are ways to earn CEUs. As the article by Roetert and Ortega was designated as a quiz article in 2019, it is likely to be read by more certified individuals than usual^26)^. Thus, translated studies can be considered valuable resources for providing information on physical literacy to Japanese people involved in sports, medicine, and science.

## Future tasks

Although the evaluation scale is still in the “draft” stage, there have been reports of its gradual use. For example, in July 2025, the author's research team asked fourth-grade students in Sagamihara City, Kanagawa, Japan to answer a questionnaire using an evaluation scale for teachers' educational references. In the future, we plan to conduct questionnaire surveys with parents to investigate the relationship between children's physical literacy and their lifestyle habits and academic performance and then provide feedback to the school. In Sagamihara City, Council Member Jin Akimoto stated at a regular council meeting in March 2025 that physical literacy has been gaining attention in other countries in recent years. He questioned the necessity of incorporating physical literacy into the city's sports policy, including the term in the Sagamihara City Sports Promotion Plan, so that the city can become a leading sports community^79)^. He requested the council's opinion on the matter, and in response, Mayor Kentaro Motomura of Sagamihara City stated,' Regarding physical literacy, we believe that being able to enjoy sports throughout one's lifetime contributes to a more enjoyable and fulfilling life. Therefore, when formulating the next Sports Promotion Plan, we should consider incorporating the concept of physical literacy.” Sagamihara City is a designated city in Japan; given that such a statement was made by a politician, it is important to closely monitor how physical literacy will be incorporated into policies.

In addition, Kanagawa prefecture will use the Physical Literacy Evaluation Scale in the prefecture's “Junior Challenge Project.” This project aims to discover and train talented athletes in Kanagawa Prefecture, and 2025 marks their fourth year. Lectures on physical literacy for elementary school students who have been scouted as athletes have already begun^80)^, and the results of a physical literacy survey of the parents of selected athletes will be presented by a research team from Senshu University at the 75th Conference of the Japan Society of Physical Education, Health and Sport Sciences in August 2025. As this research continues, we hope to gain new insights into athletes' development.

In the authors' research, this “draft version” scale is used to investigate the extent to which the concept of physical literacy is related to the Japanese elementary and middle school curriculum guidelines for physical and health education. The results were presented orally at the 74th Conference of the Japan Society of Physical Education, Health and Sport Sciences in August 2024^81)^. In relation to the above, we are currently investigating the extent to which the concept of physical literacy is covered in the textbooks used in the curriculum for JSPO's certified sports instructors. The progress in this research is expected to be discussed in the near future.

The literature search also identified studies that used physical literacy as a self-assessment tool for childcare workers, which has been translated into Japanese. This study investigates how childcare workers' physical literacy affects their activity levels and support behaviors. At the 79th Annual Meeting of the Japanese Society of Physical Fitness and Sports Medicine, held in 2024, a poster titled “Relationship between Physical Literacy and Physical Activity Support behaviors during Childcare among Childhood Educator^82)^” was presented by the Human Development and Sport Laboratory team of Juntendo University. Currently, a significant number of Japanese families send their children to day care for most of their day. Therefore, the findings of this study regarding childcare workers' physical literacy are highly meaningful. However, measures for assessing physical literacy in Japan are still in the draft stage, with no finalized versions available yet. Once reliable and valid assessment tools are developed, research and intervention studies will likely advance across various fields, including schools and sports settings. At present, as there are insufficient studies for comparative analysis with other countries, the unique strengths and weaknesses of physical literacy in Japan remain unclear. However, physical literacy questionnaires currently under consideration in Japan may contain questions that differ from those used overseas. While cultural differences exist between countries, it may be preferable to establish a certain degree of commonality in the questions used to facilitate multinational comparisons. Therefore, it is hoped that developing the Japanese version of the physical literacy assessment scale will enable physical literacy research to be put into practice in society and contribute to maintaining and improving the health of Japanese.

Thus, we gained a general understanding of Japan's current state of physical literacy. Whether this can be effectively utilized in Japanese educational and sports settings requires further investigation in each specific field. In educational settings, it is necessary to investigate the extent to which the concept of physical literacy is covered in the curriculum guidelines for school education, while, in sports settings, it is necessary to investigate the extent to which it is covered in the official sports instructor manual.

## Author contributions

KT and KS designed this study. KT and MM contributed to the data collection. KT wrote the first draft of the manuscript. All authors contributed to the revision of the manuscript and read and approved the final version.

## Conflicts of interest statement

The authors declare that there are no conflicts of interest.

## Note

The authors translated the names of Japanese publications without official English titles into English.
